# Semen Cryopreservation for Men Banking for Oligozoospermia, Cancers, and Other Conditions: 24 Years’ Experience of an Italian Bank

**DOI:** 10.3390/jcm12144657

**Published:** 2023-07-13

**Authors:** Sara Marchiani, Selene Degl’Innocenti, Sara Dabizzi, Lara Tamburrino, Maria Grazia Fino, Giulia Traini, Costanza Calamai, Mario Maggi, Linda Vignozzi, Elisabetta Baldi, Francesco Lotti

**Affiliations:** 1 Department of Experimental and Clinical Biomedical Sciences “Mario Serio”, University of Florence, 50134 Florence, Italy; sara.marchiani@unifi.it (S.M.); giulia.traini@unifi.it (G.T.); costanza.calamai@unifi.it (C.C.); mario.maggi@unifi.it (M.M.); linda.vignozzi@unifi.it (L.V.); 2 Andrology, Women’s Endocrinology and Gender Incongruence Unit, Center for Prevention, Diagnosis and Treatment of Infertility, Careggi University Hospital, 50139 Florence, Italy; deglinnocentis@aou-careggi.toscana.it (S.D.); dabizzis@aou-careggi.toscana.it (S.D.); lara.tamburrino@unifi.it (L.T.); finomg@aou-careggi.toscana.it (M.G.F.); elisabetta.baldi@unifi.it (E.B.); 3 Department of Experimental and Clinical Medicine, University of Florence, 50134 Florence, Italy

**Keywords:** semen cryopreservation, oncological patients, oligozoospermia

## Abstract

Background: Sperm cryopreservation is recommended to preserve male fertility for cancer patients or other medical conditions at risk of sperm decline. Whether motility and viability recovery rates vary depending on the medical conditions requiring cryopreservation is poorly known. We report here on the 24-year experience of our semen bank. Methods: Motility and viability recovery rates were evaluated in 1973 collections from patients with various medical conditions and 67 collections from donors, and the results were related to basal semen quality. Results: Motility and viability recovery were highly related to basal semen quality and varied between cancer and non-cancer conditions, independently of the duration of cryopreservation and patient age. In samples with a sperm number below 2 × 10^6^/mL, recovery rates approximated to zero. The highest recovery rates were found in donor collections. Cut-off values for the recovery of at least 1% motile spermatozoa were established based on initial semen quality. Conclusions: Our results indicate that the occurrence of any pathological or medical condition resulted in lower recovery rates with respect to donors, indicating that intrinsic sperm characteristics drive susceptibility to cryodamage. Established cut-off values for motility recovery can be useful for patient counseling as well as for ART laboratories to decide the type of procedure.

## 1. Introduction

The first cryobanks of human semen were born in the USA in the 1970s with the aim to guarantee fertility preservation to men undergoing vasectomy; however, the greatest development occurred in the 1980s, when donor semen began to be cryoconserved. In Europe, France established a national network of semen banks starting in 1973, gradually increasing the number of participating centers over the years. Since then, many commercial cryobanks or national services have developed worldwide to ensure fertility preservation not only for cancer patients undergoing potential gonadotoxic treatments, but also for a variety of subjects at risk for fertility deterioration. Male partners of couples included in assisted reproductive technique (ART) programs and sperm donors for use in heterologous ART cycles.

Although the use of cryopreserved spermatozoa for ART is relatively low, the reported rates of clinical pregnancy and live births do not differ, or differ slightly, from cycles with fresh semen [1,2].

Due to its particular morphological conformation, i.e., low membrane fluidity and low intracytoplasmic water content, the spermatozoon is not particularly sensitive to freeze and thaw damage. However, several adverse structural and functional effects after cryopreservation have been described in a high percentage of cryopreserved spermatozoa, including alterations in sperm membrane and mitochondrial structure as well as in motility and viability, an increase in DNA damage, and changes in genetic, epigenetic, proteomic, and metabolomic profiles [2]. The implementation of cryopreservation protocols and cryoprotectors over the years, as well as the introduction of experimental or promising approaches to prevent or reduce the damage, were mostly unsuccessful [2]. The extent of the damage depends highly on the semen quality before freezing [3,4]. In particular, our group demonstrated that sperm parameters before cryopreservation are predictive of motility recovery after thawing with high accuracy [4].

Since February 1998, the Regional Reference Center for semen cryopreservation at the Careggi University Hospital of Florence offered the possibility of preserving fertility to nearly 3000 individuals by banking over 4000 semen collections, mostly from oncological patients and oligozoospermic subjects. The latter were banking because their semen quality could decrease with time [5].

In this study, we report the 24-year experience of our bank by retrospectively summarizing, according to pathologies, the population that underwent semen cryopreservation, the duration of storage, and the reasons for sample elimination, as requested by the patient. In addition, we report data on the recovery of sperm motility and viability after thawing in relation to semen quality before freezing, updating the results of our previous study [4], which included the evaluation of 822 collections with 1973 current collections. We also performed the evaluation of sperm motility and viability recovery from semen collections derived from donors stored in our bank.

Such a long banking experience will be helpful in understanding which categories of subjects are most affected by cryodamage and whether a different susceptibility to cryodamage exists between patients with tumors and patients cryopreserving for a different reason or donors. Most importantly, we evaluated the relationship between baseline semen quality and recovery rates to understand whether the former may be used as an indication for laboratory personnel to choose the optimal method or device to adopt for cryostorage. These results may also be helpful to the clinician to provide more appropriate and individualized counseling for the patient.

## 2. Materials and Methods

### 2.1. Study Population

The study was retrospectively conducted on semen collections from 2913 patients undergoing cryopreservation at the Regional Reference Center for semen cryopreservation at the Careggi University Hospital of Florence from February 1998 to February 2023. As some patients underwent more than one semen collection to increase the number of cryopreserved straws, a total of 4006 samples have been collected. In addition, 67 semen collections from 10 donors were also collected from 2016 until now.

Before banking, all patients signed an informed consent.

Data considered for the study included age at the time of cryopreservation, indication for semen cryopreservation, pre- and post-thaw sperm progressive motility and viability, current banking status, and length of storage. Banking status is defined as ongoing storage for semen collections still banked or as eliminated for those semen collections withdrawn to be used for ART or not renewed or eliminated for a patient’s death. The length of storage represents the time in years between the date of cryopreservation and the date of thawing for each semen collection.

**Figure 1:**
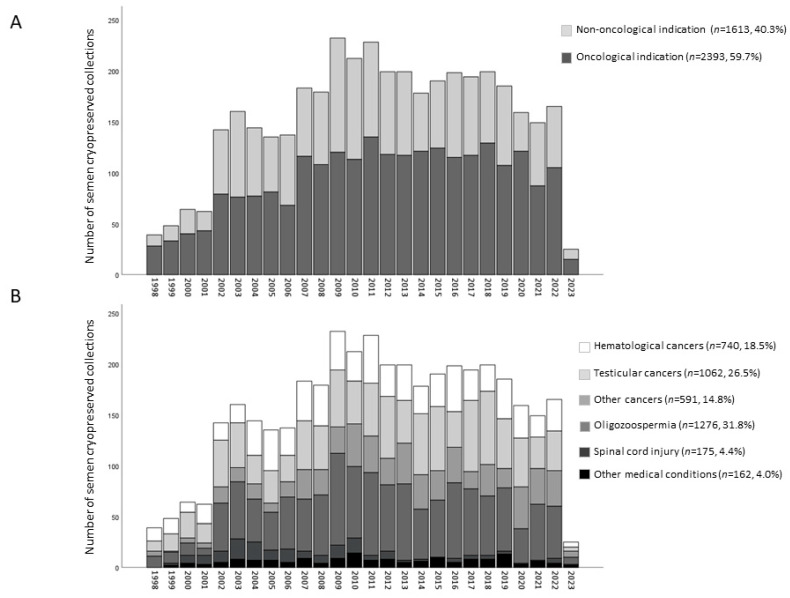
Number of semen cryopreserved collections (total, *n* = 4006) performed per year (from February 1998 to February 2023) in the different groups of subjects undergoing cryobanking, dividing by oncological and non-oncological indication (**A**) or by medical condition (**B**).

Of the 4006 samples, 2393 and 1613 were cryopreserved, respectively, for oncological and non-oncological indications (Figure 1). Oncological indications included 740 for hematological cancers, 1062 for testicular cancers, and 591 for other cancer pathologies (Figure 1). All cancer patients cryopreserved sperm before initiation of the antineoplastic treatment. In the case of testicular cancer, the majority of patients underwent cryopreservation after orchiectomy.

Among non-oncological indications, 1276 were cryopreserved for oligozoospermia, 175 for spinal cord injury, and 162 for other medical conditions (including transgender subjects, men affected by varicocele, hypogonadism, or multiple sclerosis, and Klinefelter or men with a Y chromosome deletion) (Figure 1).

Evaluation of the recovery of sperm progressive motility and viability after thawing was performed only on discarded collections (*n* = 1977). Semen samples with baseline 0% progressive motility (*n* = 256) were not considered in the statistical analysis. To eliminate possible bias in sperm motility and viability assessment after thawing due to the low number of spermatozoa at baseline, the data of patients with sperm basal concentrations of <2 × 10^6^/mL (*n* = 927, of which discarded collections *n* = 239) were analyzed and reported separately. The final number of collections on which we calculated post-thawing recovery was 1482.

### 2.2. Semen Cryopreservation

Briefly, semen samples were collected on the same day of cryopreservation by masturbation in the laboratory. In exceptional cases, semen collection was performed at home or inpatient care, and, for most spinal cord injury patients, it was performed by penile vibratory stimulation or electroejaculation. With the exception of spinal cord injury patients, all subjects were asked to observe 2–7 days of sexual abstinence.

After semen analysis (see below), whole semen samples were diluted 1:1 (vol:vol) by the drop-wise addition of test yolk buffer (TYB, provided by FUJIFILM Italia S.p.A. (Milan, Italy)) containing egg yolk (20%), glycerol (12% *v*/*v*), and gentamycin (10 μg/mL) and, after equilibration at room temperature for 5–10 min, sperm were loaded in 500 µL high-security sperm straws (Cryo Bio System, L’Aigle, France). Then, samples were frozen in liquid nitrogen tanks by a manually controlled freezing procedure that involved first exposing the straws for 8 min to liquid nitrogen vapors and, finally, placing them into liquid nitrogen.

Thawing was carried out by keeping the straws for 15 min at 37 °C in a dry incubator before observation.

### 2.3. Pre- and Post-Cryopreservation Semen Analysis

Semen analysis was performed according to WHO guidelines [6,7,8]. Pre- and post-cryopreservation sperm motility was assessed by light microscopy, according to WHO criteria [6,7,8]. Briefly, sperm motility was evaluated by a direct optical microscope using a 40X objective with a 37 °C heated plate (Nikon Eclipse Ci). Sperm concentration was evaluated by an improved Neubauer chamber after dilution in formalin containing buffer. Sperm concentration for samples showing a low number of spermatozoa/field were reported as <2 × 10^6^/mL according to WHO [7,8]. The percentages of progressive, non-progressive, and immotile spermatozoa were evaluated on 200 spermatozoa/sample. Sperm viability was evaluated by using an eosin test according to the WHO manual [6,7,8].

The occurrence of leukocytes in semen was determined by deposing 10 μL of semen on pre-stained slides Testsimplets^®^ (AB Analitica, Padua, Italy) and subsequent evaluation with an optical microscope at 1000X.

The Regional Reference Center for semen cryopreservation at the Careggi University Hospital of Florence has been participating in the UK-NEQAS (United Kingdom National External Quality Assessment Service) external quality control program for semen analysis since 2005. The mean (± SD) percent biases of the laboratory for the year 2022 were for total and progressive motility 2.9 (±8.5) and 8.5 (±11.1), respectively, and for a sperm concentration of 14.5 (±9.7) (*n* = 16, data from UK-NEQAS). Sperm morphology data were not analyzed because the method of assessing sperm morphology varied during the study years (according to the fourth edition of the WHO manual [6], and after that, using strict criteria as indicated in the fifth and sixth editions [7,8]).

### 2.4. Statistical Analysis

Statistical analysis was performed using the Statistical Package for the Social Sciences version 28.0 (SPSS) for Windows.

By using the Kolmogorov–Smirnov test, we verified that the data was not normally distributed. Data are shown as median values and interquartile ranges. Statistically significant differences between pre- and post-cryopreservation sperm motility and viability were assessed using the nonparametric Kruskal–Wallis test. The Mann–Whitney test was used for comparisons among groups. Correlations between pre- and post-cryopreservation parameters were assessed by Spearman’s correlation test.

ROC analysis was used as a binary classifier system to identify the accuracy of the pre-cryopreservation semen parameters, predicting the post-thawing recovery of at least 1% motility. Such a value was arbitrary chosen, considering the minimum requirement to find at least one motile spermatozoon after observation of 100 spermatozoa in post-thawing samples by an embryologist.

*p* < 0.05 was considered statistically significant.

## 3. Results

### 3.1. Characteristics of Patients, Disposal, and Duration of Semen Collections

Figure 1 summarizes the number of collections by year from February 1998 to February 2023. It can be noted that starting in the 2000s, the number of cryopreservations/year increased, reaching about 200 collections/year from 2010 onward. Most of the banked semen samples were derived from oncological patients (59.7%, Figure 1A), and among them, the most represented were testicular cancers (26.5%, Figure 1B). Among non-oncological conditions, 31.8% of samples were cryopreserved for oligozoospermia (Figure 1B). These proportions among the various pathological categories are maintained over time (Figure 1A,B).

**Figure 2:**
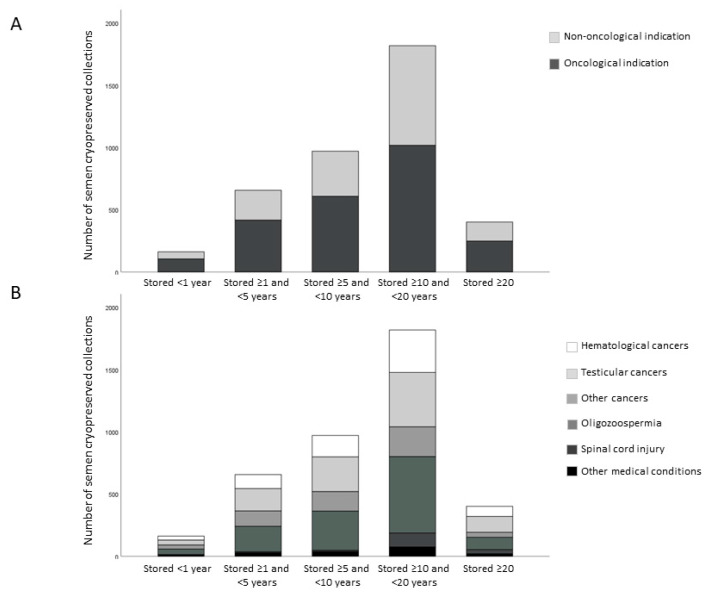
Number of semen collections according to the duration period of cryopreservation in the different groups of subjects undergoing cryobanking, divided into oncological and non-oncological indications (**A**) or by medical condition (**B**).

Concerning the duration period of ongoing semen cryopreserved collections, 7.5% (*n* = 160) are stored for less than a year, 30.0% (*n* = 638) from one to 5 years, 33.0% (*n* = 702) from 5 to 10 years, 27.1% (*n* = 576) from 10 to 20 years, and 2.4% (*n* = 50) for more than 20 years. Figure 2 shows the number of samples in oncological and non-oncological subjects (panel A) as well as in different pathological conditions (panel B) according to the duration of cryopreservation.

In Table 1, the median values of age, sexual abstinence, and semen parameters (at the time of semen collection and cryopreservation) of men with a sperm concentration of ≥2 × 10^6^/mL are reported. Non-oncologic subjects show an older age compared to oncologic ones, whereas total sperm count and the percentage of sperm progressive motility and viability at baseline are significantly higher in oncological patients compared to non-oncological patients (Table 1). In particular, among oncological patients, testicular cancers evidenced lower median values of sperm count, progressive motility, and viability. Among non-oncological subjects, a higher sperm count but lower sperm motility and viability are observed in men with spinal cord injuries. As expected, oligozoospermic subjects exhibit poor semen quality.

Table 2 reports the age and semen characteristics of subjects with a sperm concentration of <2 × 10^6^/mL, considered a separate cohort in the statistical analysis.

Of the 4006 collections banked, 1973 were eliminated for various reasons, including use for ART procedures, patient death, or because the patient did not renew the maintenance of the collection (Table 3). As can be observed, most collections were eliminated due to patients not-renewal, both in oncological and non-oncological patients, and the percentage of patients used for ART procedures was similar in the two groups (Table 3). Of note, in the group of spinal cord injuries, the percentage of samples used for ART treatment is considerably higher (26.5%) compared to overall values (5.5%).

The median value of the duration of cryopreservation for non-oncological discarded collections is lower (3.6 [IQR: 2.2–6.4]) than for oncological ones (4.1 [IQR: 2.6–7.2]); among the latter, testicular cancers is the category that preserved semen for the longest time (5.1 [IQR: 3.4–9.8]).

### 3.2. Effect of Cryopreservation on Sperm Parameters

**Figure 3:**
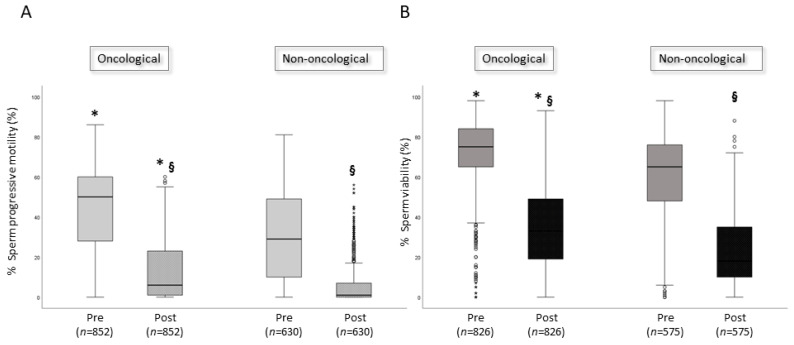
Box plots representing sperm pre- and post-cryopreservation progressive motility (**A**) and viability (**B**) in the group of oncological and non-oncological indications with sperm concentrations of ≥2 × 10^6^/mL at baseline. * *p* < 0.05 vs. Non-oncological indication, Mann–Whitney test; § < 0.05 vs. Pre-cryopreservation, Wilcoxon test.

**Figure 4:**
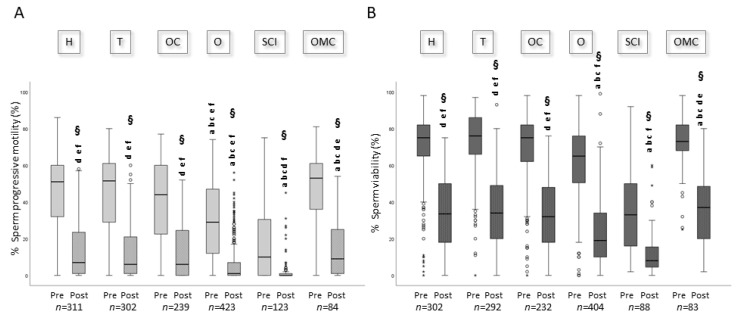
Box plots representing sperm pre- and post-cryopreservation progressive motility (**A**) and viability (**B**) in the different groups of subjects undergoing cryobanking with sperm concentrations of ≥2 × 10^6^/mL at baseline. H: Hematologic cancers; T: Testicular cancers; OC: Other cancers; O: Oligozoospermia; SCI: Spinal cord injury; OMC: Other medical conditions. a: *p* < 0.05 vs. H; b: *p* < 0.05 vs. T; c: *p* < 0.05 vs. OC; d: *p* < 0.05 vs. O; e: *p* < 0.05 vs. SCI; f: *p* < 0.05 vs. OMC, Mann–Whitney test. § < 0.05 vs. Pre-cryopreservation, Wilcoxon test.

A straw from each eliminated collection was used to determine the percentage of sperm with progressive motility and viability after freezing and thawing. Median values of sperm pre- and post-cryopreservation progressive motility (A) and viability (B) for samples with concentration ≥2 × 10^6^/mL at baseline, in the groups of oncological and non-oncological subjects and in the other conditions, are reported in Figure 3 and Figure 4, respectively. As observed, both sperm parameters are significantly lower post-freezing and thawing for all categories of subjects (Figure 3 and Figure 4). In particular, the recovery rates in oncological patients are 15.7% [IQR: 3.6–43.3] for motility and 46.9% [IQR: 28.2–64.0] for viability. Recovery rates are significantly lower in non-oncological subjects (6.3% [IQR: 0.0–24.1] and 33.3% [IQR: 19.0–52.1] for sperm motility and viability, respectively, *p* < 0.05)). Among the latter group, oligozoospermic and spinal cord injury patients show the worst recovery for both motility (6.0% [IQR: 0.0–21.7] and 1.0% [IQR: 0.0–13.1]) and viability (31.6% [IQR: 18.3–50.7] and 27.8% [IQR: 14.3–46.7]). Dividing subjects into three groups according to their age at the time of cryopreservation (<18, 18–45, and >45), no differences are observed regarding the recovery rates of motility and viability in all the patients’ categories. When we divided our patients into two groups based on the presence of leucocytes in semen, we found that samples with leucocytes show a statistically significant lower progressive motility recovery relative to those without leucocytes (2.9 [IQR: 0.0–22.7] vs. 7.8 [IQR: 0.0–30.0], *p* = 0.006). The overall median recovery values for motility and viability in our patients (oncological and non-oncological) were 11.9% [IQR: 0.0–35.6] and 41.1% [IQR: 24.0–60.0], respectively.

The recovery of progressive motility for semen samples with a sperm concentration of <2 × 10^6^/mL at baseline is 0% for all categories.

We also evaluated the percentages of pre- and post-cryopreservation sperm motility and viability in samples from 10 donors stored in our bank (Table 4). As can be observed, the median values of both parameters are higher compared to oncological and non-oncological patients (Figure 3), with recovery rates of 61.5% [IQR: 46.2–77.9] for motility and 63.8% [IQR: 53.4–78.8] for viability (*n* = 67 donor semen collections).

**Figure 5:**
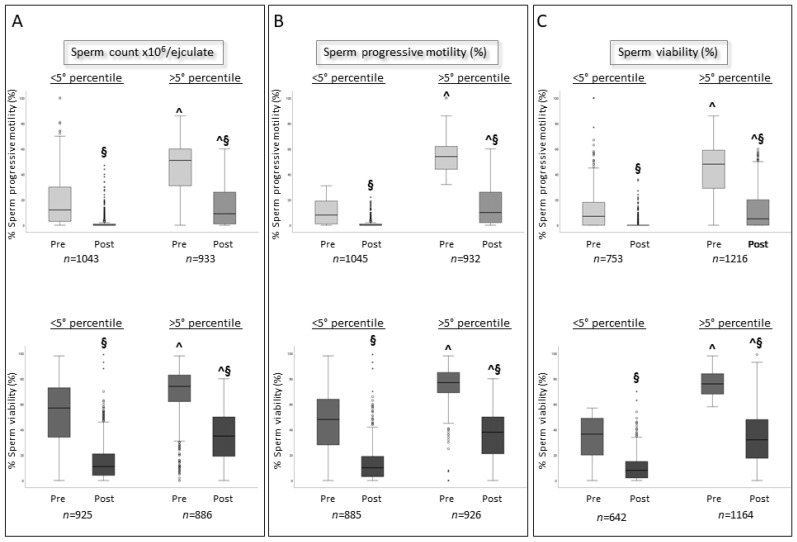
Box plots representing pre- and post-cryopreservation sperm progressive motility (upper panels) and viability (lower panels) divided according to the 5° percentile of WHO 2010 reference values of basal sperm count (**A**), progressive motility (**B**) and viability (**C**). ^ *p* < 0.05 vs. <5° percentile, Mann–Whitney test. § < 0.05 vs. Pre-cryopreservation, Wilcoxon test.

When subjects were divided according to the 5° percentile of the WHO 2010 reference values, post-cryopreservation motility was significantly lower in samples from men with sperm number (Figure 5A, upper panel), progressive motility (Figure 5B, upper panel), and viability (Figure 5C, upper panel) below the 5° percentile at baseline, regardless of the baseline condition. Similar results are found for sperm viability (Figure 5A–C, lower panels). Such data confirm those obtained in our previous study [4]. In addition, statistically significant positive correlations are found between pre-cryopreservation sperm parameters and the percentages of viable and progressively motile spermatozoa after cryopreservation in all categories of subjects (Table 5). Lower or no associations were observed for donor collections (Table 5), probably because of the low number of cases. No association is observed between the recovery of sperm progressive motility and viability with storage time.

**Figure 6:**
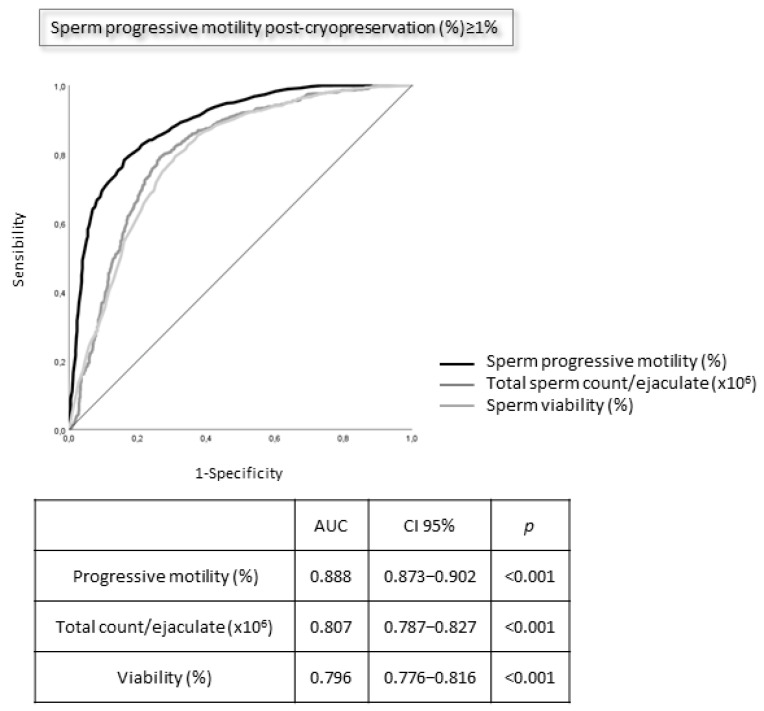
Receiver operating characteristic (ROC) curves for the prediction of sperm progressive motility (≥1%) post-cryopreservation, comparing baseline sperm progressive motility, total sperm count/ejaculate, and sperm viability. AUC: area under the curve; CI: 95% confidence interval; *p*: statistical significance.

ROC curves of all basal sperm characteristics (total sperm count, progressive motility, and viability) predict post-cryopreservation sperm progressive motility of at least 1% with good accuracy (Figure 6). In particular, higher AUC values were observed for basal progressive motility. Cut-off values for at least 1% progressive motility recovery were 21 × 10^6^/ejaculate (sensitivity 80% and specificity 80%) for sperm number, 30% (sensitivity 76% and specificity 95%) for sperm progressive motility, and 63% (sensitivity 76% and specificity 72%) for sperm viability.

## 4. Discussion

Sperm cryopreservation is widely used nowadays to ensure fertility preservation in men at high risk for fertility decline and to collect semen from donor men for heterologous ART procedures. Despite such a large use of the procedure, at present there are no key performance indicators (KPIs) for sperm banks regarding motility and viability recoveries after thawing. In addition, it is poorly known whether recoveries vary among the different pathologies or conditions requiring sperm banking.

We show here, by evaluating a large series of samples, that the percentage decrease in motility post-thawing is higher in patients cryopreserving for non-oncological problems with respect to oncological ones. Although non-oncological patients showed, on average, lower sperm number, progressive motility, and viability at baseline (Table 2), the percentage decrease of motility and viability due to cryodamage was expected to be similar. In particular, among non-oncological patients, oligozoospermic and spinal cord injured patients showed the lowest recovery rates for both motility and viability, suggesting a higher susceptibility to cryodamage. Although we did not experience a decline in median sperm concentration/total number during our 24 years’ experience in our cohort of patients, this result is particularly alarming in light of the global decline in sperm counts reported in recent decades in the general male population [9].

In addition, we found that the overall recovery rates of motility and viability in our cohort of patients cryopreserving for oncological and non-oncological reasons were considerably lower compared to recovery rates obtained in 67 semen collections from 10 sperm donors (selected on the basis of strict regulations in order to avoid pathological conditions), indicating that the presence of any pathological condition increases sperm susceptibility to cryodamage. This result reinforces the concept that intrinsic characteristics of spermatozoa impact the susceptibility to cryopreservation damage [2]. Likely, this higher susceptibility observed in subjects with pathological conditions might be due to the presence of an inflammatory status leading to high semen levels of ROS or other inflammatory mediators [10,11,12,13]. This hypothesis is confirmed by the fact that, dividing our caseload into two groups based on the presence or absence of leucocytes in semen, the recovery of sperm progressive motility is significantly lower in samples with leucocytes, which could represent a source of ROS. Such substances may impair sperm motility and viability during the semen preparation and freezing and thawing procedures, as our study clearly shows that length of storage or the patient’s age do not affect the recovery rates. There are several possibilities to improve motility and viability recovery after cryopreservation. Among these, sperm selection by swim-up or Density Gradient Centrifugation, which eliminates seminal fluid and dead and immotile spermatozoa, has been reported to improve the recovery after cryopreservation [2,14], indicating that, when possible, such strategies should be applied especially for semen samples with lower baseline quality. In this respect, our results, showing that samples with at least one parameter at baseline semen analysis below the fifth percentile of reference values of WHO [7] show worse motility and viability recovery, can be of help in identifying those samples where an additional selection procedure may improve cryopreservation recovery rates.

An important result of our study regards the fact that in subjects presenting very low semen quality (with a sperm number below 2 million/mL), the median recovery rate for sperm motility was 0%. In most of these samples, we were unable to find any motile sperm by observing 200 spermatozoa, despite a median recovery of viability of 13%. This result indicates that the eventual use of assisted reproduction will require, most likely, additional procedures by the embryologist in order to recognize and select a viable spermatozoon based on the addition of chemical substances or the employment of biophysical approaches during the ICSI procedure [14,15,16]. Recent findings demonstrating that the use of special devices (such as SpermVD^®^, Sperm Vitrification Device) to vitrify a small number of motile spermatozoa selected one by one by an ICSI pipette produces very good recovery rates of number and motility and facilitates their use in ART laboratories [17,18], open new perspectives for cryopreservation of samples with low semen quality.

Interestingly, the type of cancer does not influence post-cryopreservation motility and viability recoveries, despite the slightly lower baseline semen parameters observed in testicular cancer patients. Since most testicular cancer patients perform cryopreservation before undergoing surgery, we expected a lower recovery rate because of the possible direct exposure of germ cells to the tumoral environment. Overall, our findings reassure us about the possibility to perform semen cryopreservation before surgery in testicular cancer patients.

Regarding the usage rate of cryopreserved spermatozoa for ART procedures, our results, in line with the current literature (reviewed in 2), indicate that only a small percentage of cancer patients retrieve their device. Indeed, most cancer patients recover spermatogenesis after chemo- or radio-therapies [19,20]. Despite this fact, cryopreservation must be offered to all cancer patients before the initiation of therapies because recovery of fertility is not predictable and also because patients are advised not to attempt natural conception before two years after the last gonadotoxic treatment. Interestingly, the highest usage rate in our cohort was observed in spinal cord injured patients, probably because the procedure to obtain fresh semen collection is invasive and difficult to perform.

ROC analysis demonstrates that cut-off values can be established to estimate motility recovery of at least 1% on the basis of initial semen quality with high accuracy. Therefore, initial semen quality may be helpful for the clinician to provide appropriate counseling to the patient regarding the type of procedure to be used in future ART treatments, as well as for the embryologist to decide the number of straws needed for the procedure.

In Italy, a KPI for progressive motility recovery has been established at least 50% by the Conference of Regions and Autonomous Provinces [21] for donor sperm collections, but, as previously mentioned, at present there are no KPIs regarding semen banks for oncological and non-oncological indications. Establishing KPI for semen cryopreservation can help clinicians and embryologists to choose the best option for ART procedures. Our results suggest that KPI for motility recovery should be established both on the basis of initial semen quality and the clinical condition deserving cryopreservation. In our bank, established KPIs for donor collections have been achieved, indicating overall good performance. Our results, along with those of other semen banks, could be useful for determining KPI values for non-donor collections.

Our study has the strengths of a large number of semen collections from different types of clinical conditions as well as a long-lasting experience. However, this study has the limitation of a lack of reproductive outcomes from ART centers that use cryopreserved semen from our bank due to two different reasons: (i) our semen bank is not connected with an ART center; and (ii) we distribute cryopreserved samples to several Italian and foreign centers that do not share these data due to the politics of the individual centers and privacy reasons.

## 5. Conclusions

In conclusion, our results suggest that the conventional method for semen cryopreservation allows the recovery of motile sperm after thawing when basal semen quality is good, although the concomitant presence of a pathological condition results in lower recovery rates with respect to donors. Therefore, future studies should be devoted to investigating strategies to improve recovery rates in such medical conditions.

## Figures and Tables

**Table 1:** Median [interquartiles] of age, sexual abstinence, and semen parameters at the time of cryopreservation in the different groups of subjects, considering only semen samples with sperm concentrations of ≥2 × 10^6^/mL.

	Age (Years)	Sexual Abstinence (Days)	Volume (mL)	pH	Total Sperm Count/ Ejaculate (×10^6^)	Progressive Motility (%)	Viability (%)
**Total**	33.0 [26.0–38.0] *(n* = 3073)	4.0 [3.0–6.0] (*n* = 2819)	2.8 [1.9–4.0] (*n* = 3073)	7.6 [7.4–7.8] (*n* = 3072)	65.6 [22.8–177.6] (*n* = 3073)	43.0 [23.0–57.0] (*n* = 3073)	74.0 [61.0–83.0] (*n* = 3072)
**Oncological** **indication**	31.0 [25.0–37.0] * (*n* = 2025)	4.0 [3.0–6.0] (*n* = 1891)	2.9 [2.0–4.0] * (*n* = 2025)	7.6 [7.4–7.8] * (*n* = 2024)	82.5 [27.8–194.5] * (*n* = 2025)	48.0 [29.0–59.0] * (*n* = 2025)	76.0 [65.0–85.0] * (*n* = 2024)
**Non-** **oncological** **indication**	35.0 [29.0–40.0] (*n* = 1048)	4.0 [3.0–5.0] (*n* = 928)	2.7 [1.6–4.0] (*n* = 1048)	7.6 [7.4–7.8] (*n* = 1048)	46.0 [16.7–133.5] (*n* = 1048)	31.0 [15.3–50.0] (*n* = 1048)	68.0 [50.0–80.0] (*n* = 1048)
**H**	30.0 [23.0–37.0] ^c,d,e,f^ (*n* = 650)	4.0 [3.0–7.0] ^b,d,e,f^ (*n* = 598)	2.7 [1.7–3.8] ^b,d,e,f^ (*n* = 650)	7.6 [7.4–7.8] (*n* = 649)	103.8 [37.4–240.8] ^b,d,e,f^ (*n* = 650)	48.0 [29.0–58.0] ^d,e,f^ (*n* = 650)	75.0 [64.0–83.0] ^b,d,e,f^ (*n* = 649)
**T**	31.0 [26.0–36.0] ^c,d,e,f^ (*n* = 840)	4.0 [3.0–5.0] ^a,c,d,e,f^ (*n* = 791)	3.0 [2.0–4.2] ^a,c,d,e,f^ (*n* = 840)	7.6 [7.4–7.6]^c^ (*n* = 840)	56.1 [20.7–129.8] ^a,c,d,e,f^ (*n* = 840)	49.5 [30.0–60.0 ^d,e,f^ (*n* = 840)	78.0 [68.0–86.0] ^a,c,d,e,f^ (*n* = 840)
**OC**	32.0 [23.0–42.0] ^a,b,d,e^ (*n* = 535)	4.0 [3.0–6.0] ^d,e,f^ (*n* = 502)	2.8 [1.8–3.9] ^b,d,e,f^ (*n* = 535)	7.6 [7.4–7.8] ^d,e,f^ (*n* = 535)	114.0 [33.8–260.0] ^b,d,e,f^ (*n* = 535)	46.0 [28.0–58.0] ^d,e,f^ (*n* = 535)	76.0 [64.0–83.0] ^b, d,e,f^ (*n* = 535)
**O**	35.0 [29.0–40.0] ^a,b,c^ (*n* = 733)	4.0 [3.0–5.0] ^a,b,c,e,f^ (*n* = 714)	3.0 [2.0–4.0] ^a,b,c,e,f^ (*n* = 733)	7.6 [7.4–7.8] ^c^ (*n* = 733)	30.7 [13.0–74.7] ^a,b,c,e,f^ (*n* = 733)	31.0 [16.5–49.5] ^a,b,c,e,f^ (*n* = 733)	70.0 [57.0–80.0] ^a,b,c,e,f^ (*n* = 733)
**SCI**	34.0 [30.0–38.0] ^a,b,c^ (*n* = 158)	6.0 [4.0–10.0] ^a,b,c,d,f^ (*n* = 60)	2.0 [1.0–3.0] ^a,b,c,d,f^ (*n* = 158)	7.6 [7.4–7.8] ^c^ (*n* = 158)	246.5 [52.7–576.9] ^a,b,c,d,f^ (*n* = 158)	17.0 [5.8–31.0] ^a,b,c,d,f^ (*n* = 158)	30.0 [15.0–50.0] ^a,b,c,d,f^ (*n* = 158)
**OMC**	34.0 [27.0–43.0] ^a,b^ (*n* = 157)	4.0 [3.0–5.3] ^a,b, c,d,e^ (*n* = 154)	2.5 [1.6–3.6] ^a,b,c,d,e^ (*n* = 157)	7.6 [7.4–7.6] ^c^ (*n* = 157)	112.0 [46.8–236.0] ^a,b,c,d,e^ (*n* = 157)	51.0 [32.5–60.0] ^a,b,c,d,e^ (*n* = 157)	73.0 [65.0–83.5] ^a,b,c, d,e^ (*n* = 157)

* *p* < 0.05 vs. Non-oncological indication; ^a^ *p* < 0.05 vs. Hematologic cancers (H); ^b^ *p* < 0.05 vs. Testicular cancers (T); ^c^ *p* < 0.05 vs. Other cancers (OC); ^d^ *p* < 0.05 vs. Oligozoospermia (O); ^e^ *p* < 0.05 vs. Spinal cord injury (SCI); ^f^ *p* < 0.05 vs. Other medical conditions (OMC).

**Table 2:** Median [interquartiles] of age, sexual abstinence, and semen parameters at the time of cryopreservation in the different groups of subjects, considering only semen samples with sperm concentrations of <2 × 10^6^/mL.

	Age (Years)	Sexual Abstinence (Days)	Volume (mL)	pH	Progressive Motility (%)	Viability (%)
**Total**	33.0 [28.0–39.0] (*n* = 927)	4.0 [3.0–5.0] (*n* = 876)	3.0 [2.0–4.4] (*n* = 927)	7.6 [7.4–7.6] (*n* = 927)	7.0 [1.0–16.0] (*n* = 926)	46.0 [24.0–65.0] (*n* = 926)
**Oncological** **indication**	31.0 [25.0–36.0] * (*n* = 364)	4.0 [3.0–5.0] (*n* = 354)	2.9 [2.0–4.0] * (*n* = 364)	7.6 [7.4–7.8] * (*n* = 364)	6.0 [1.0–15.0] (*n* = 363)	46.0 [25.0–66.0] (*n* = 363)
**Non-oncological** **indication**	35.0 [30.0–40.0] (*n* = 563)	4.0 [4.0–5.0] (*n* = 522)	3.2 [2.2–4.7] (*n* = 563)	7.6 [7.4–7.6] (*n* = 563)	7.0 [1.0–16.0] (*n* = 563)	46.0 [21.0–65.0] (*n* = 563)
**H**	28.0 [20.5–34.0]^c,b,d,e,f^ (*n* = 90)	3.0 [3.0–5.0] ^d,e,f^ (*n* = 87)	2.5 [1.8–3.8] ^b,d,e,f^ (*n* = 90)	7.6 [7.4–7.8] (*n* = 90)	4.0 [0.0–9.0] ^d,e,f^ (*n* = 90)	34.5 [16.0–65.8] ^e,f^ (*n* = 90)
**T**	31.0 [26.0–36.0] ^a,d,e,f^ (*n* = 222)	4.0 [3.0–5.0] ^d,e,f^ (*n* = 216)	3.0 [2.0–4.2] ^a,c,d,e^ (*n* = 222)	7.6 [7.4–7.6]^c^ (*n* = 222)	7.0 [2.0–17.0] ^c,e,f^ (*n* = 222)	49.0 [30.0–67.0] ^e,f^ (*n* = 222)
**OC**	33.0 [27.0–39.0] ^a^ (*n* = 56)	4.0 [3.0–5.0] ^d,e,f^ (*n* = 55)	2.4 [1.6–4.0] ^b,d,e,f^ (*n* = 56)	7.6 [7.4–7.8] ^b,d,e,f^ (*n* = 56)	4.5 [0.3–11.8] ^d,e,f^ (*n* = 56)	44.5 [21.0–64.5] ^e,f^ (*n* = 56)
**O**	35.0 [30.0–40.0] ^a,b,e^ (*n* = 543)	4.0 [4.0–5.0] ^a,b,c,e,f^ (*n* = 513)	3.2 [2.3–4.8] ^a,b,c^ (*n* = 543)	7.6 [7.4–7.6] ^c^ (*n* = 543)	8.0 [2.0–17.0] ^a,c,e,f^ (*n* = 543)	47.0 [25.0–65.0] ^e,f^ (*n* = 543)
**SCI**	38.0 [31.5–38.0] ^a,b,d^ (*n* = 17)	11.0 [6.5–30.0] ^a,b,c,d^ (*n* = 6)	2.1 [1.3–4.0] ^a,b,c^ (*n* = 17)	7.4 [7.0–7.6] ^c^ (*n* = 17)	0.0 [0.0–7.5] ^a,b,c,d^ (*n* = 17)	9.0 [0.0–39.5] ^a,b,c,d^ (*n* = 17)
**OMC**	32.0 [21.0–32.0] ^a,b^ (*n* = 5)	5.0 [4.0–] ^a,b,c,d^ (*n* = 5)	3.5 [0.6–4.7] ^a,c^ (*n* = 5)	7.4 [7.4–7.4] ^c^ (*n* = 5)	0.0 [0.0–19.5] ^a,b,c,d^ (*n* = 5)	6.0 [0.0–36.8] ^a,b,c,d^ (*n* = 5)

* *p* < 0.05 vs. Non-oncological indication; ^a^ *p* < 0.05 vs. Hematologic cancers (H); ^b^ *p* < 0.05 vs. Testicular cancers (T); ^c^ *p* < 0.05 vs. Other cancers (OC); ^d^ *p* < 0.05 vs. Oligozoospermia (O); ^e^ *p* < 0.05 vs. Spinal cord injury (SCI); ^f^ *p* < 0.05 vs. Other medical conditions (OMC).

**Table 3:** Number of cryopreserved semen collections (frequency, %) used for ART, not renewed or eliminated due to patient death, and divided by oncological or non-oncological indication.

	All		
	Used for ART	No Renewal	Death
**Total (*n* = 1973)**	109 (5.5%)	1722 (87.3%)	142 (7.2%)
**Oncological indication (*n* = 994)**	31 (3.1%)	826 (83.1%)	137 (13.8%)
**Non-oncological indication (*n* = 979)**	78 (8.0%)	896 (91.5%)	5 (0.5%)

**Table 4:** Median [interquartiles] of sperm pre- and post-cryopreservation progressive motility and viability in 10 banked donors.

	Pre-Cryopreservation Progressive Motility (%)	Post-Cryopreservation Progressive Motility (%)	Pre-Cryopreservation Viability (%)	Post-Cryopreservation Viability (%)
**All Donors**	59.0 [55.0–66.0] (*n* = 67)	38.0 [28.0–46.0] (*n* = 67)	84.0 [78.0–90.0] (*n* = 67)	54.0 [45.0–64.0] (*n* = 67)

*n*: number of collections.

**Table 5:** Correlations between pre-cryopreservation sperm parameters (total sperm count/ejaculate, progressive motility, and viability) and post-cryopreservation sperm parameters (progressive motility and viability) were calculated in all patients, oncological and non-oncological groups, and donors. R: Spearman’s rank correlation coefficient, *p*: statistical significance, *n*: number of semen collections. ns: not significant.

		Pre-Cryopreservation Parameters			
			Total Sperm Count/ Ejaculate (×10^6^)	Progressive Motility (%)	Viability (%)
**Post-cryopreservation parameters**	**All patients**	**Progressive motility (%)**	R = 0.6, *p* < 0.001 (*n* = 1973)	R = 0.8, *p* < 0.001 (*n* = 1973)	R = 0.6, *p* < 0.001 (*n* = 1969)
		**Viability (%)**	R = 0.6, *p* < 0.001 (*n* = 1811)	R = 0.7, *p* < 0.001 (*n* = 1811)	R = 0.6, *p* < 0.001 (*n* = 1806)
	**Oncological indication**	**Progressive motility (%)**	R = 0.7, *p* < 0.001 (*n* = 995)	R = 0.7, *p* < 0.001 (*n* = 995)	R = 0.7, *p* < 0.001 (*n* = 948)
		**Viability (%)**	R = 0.6, *p* < 0.001 (*n* = 948)	R = 0.7, *p* < 0.001 (*n* = 948)	R = 0.6, *p* < 0.001 (*n* = 945)
	**Non-oncological indication**	**Progressive motility (%)**	R = 0.5, *p* < 0.001 (*n* = 978)	R = 0.7, *p* < 0.001 (*n* = 978)	R = 0.5, *p* < 0.001 (*n* = 976)
		**Viability (%)**	R = 0.5, *p* < 0.001 (*n* = 863)	R = 0.6, *p* < 0.001 (*n* = 863)	R = 0.6, *p* < 0.001 (*n* = 861)
	**Donors**	**Progressive motility (%)**	R = 0.1, *p* = ns (*n* = 67)	R = 0.2, *p* < 0.05 (*n* = 67)	R = 0.3, *p* < 0.05 (*n* = 67)
		**Viability (%)**	R = 0.1, *p* = ns (*n* = 67)	R = 0.1, *p* = ns (*n* = 67)	R = 0.3, *p* < 0.05 (*n* = 67)

## Data Availability

Data are not available due to privacy or ethical restrictions.
